# MRI evaluation of meniscal anatomy: which parameters reach the best inter-observer concordance?

**DOI:** 10.1007/s11547-022-01527-z

**Published:** 2022-07-14

**Authors:** Dario Grasso, Aroa Gnesutta, Marco Calvi, Marta Duvia, Maria Giovanna Atria, Angelica Celentano, Leonardo Callegari, Eugenio Annibale Genovese

**Affiliations:** 1 University of Insubria, 21100 Varese, Italy; 2 ASST-Settelaghi, Ospedale di Circolo e Fondazione Macchi, 21100 Varese, Italy; 3 Medical Clinical Institute Intermedica - Columbus, Milan, Italy; 4 University of Insubria, 21100 Varese, Italy

**Keywords:** Observer variation, Magnetic resonance imaging, Meniscus, Knee joint

## Abstract

**Purpose:**

The aim of the study is to evaluate which MRI parameters achieve the best degree of inter-individual concordance in the description of meniscal fibrocartilage, regarding its morphology, signal and position.

**Materials and methods:**

Eighty-nine knee MRIs were included in the study, retrospectively re-evaluated by three radiologists who completed a binary report (normal/abnormal) describing the meniscus signal, position relative to the tibial plateau margin and morphology. The inter-individual concordance value was calculated using Cohen's test.

**Results:**

We obtained different inter-individual concordance values according to the parameters considered. The concordance was poor in the description of the meniscal position relative to the tibial plateau margin (average *k* = 0.6); the result was comparable in the description of the meniscal morphology (average *k* = 0.56). The best results were obtained with the meniscal signal analysis (average *k* = 0.8).

**Conclusion:**

To the best of our knowledge, there are no studies in the literature assessing the concordance between multiple readers in the description of the parameters we studied. The results we obtained suggest that the most reliable parameter for describing meniscal fibrocartilage is its signal intensity, whereas morphology and position may lead to different interpretations that are not always unequivocal.

## Introduction

The menisci are semicircular, intra-articular, fibrocartilaginous structures act to disperse the weight of the body and reduce friction during movement [1, 2].

To our knowledge, meniscal pathology may predispose the onset of knee diseases, which are highly prevalent in the general population [3, 4]. Meniscal lesions contribute to the progressive loss of cartilage and cause the development of osteoarthritis [5, 6].

The meniscus may have alterations in the morphology, position and intensity of the signal. The abnormal morphology can be caused by congenital anatomical variants including the discoid meniscus, anterior or posterior megacorn, and by degenerative changes such as meniscal thinning.

Fibrocartilaginous menisci, normally, are localized in knee joint, within tibio-femoral compartments. There could be an alteration of position with extrusion of meniscus, secondary to overload of tibio-femoral compartment or to meniscal fragment dislocation.

In physiological conditions, menisci should have homogeneous low signal intensity at MRI imaging; alterated signal intensity is commonly classified into three types. Type I shows focal degenerative phenomena starting from the central region of the fibrocartilage.

Type II represents the extension without involvement of the articular sides of the meniscus.

Type III, instead, affects at least one joint side of the meniscus and it is considered the most significant and symptomatic.

Horizontal lesions mostly engage people over 40 years of age and represent the consequence of degenerative changes, while radial tears, in particular lateral meniscus, interest younger population and they are often caused by traumatic events [7].

Magnetic resonance imaging (MRI) is the most accurate technique for the diagnosis of knee pathologies, [8–10], and it is considered the gold standard examination for evaluation of meniscal derangements [11].

Therefore, the correct identification of the signal anomalies, morphology as well as position represents a primary step in the correct knee assessment. However, meniscus extrusion and size, as well as its signal alterations, may be difficult to identify [12–14].

The purpose of this study is to determine the inter-individual concordance between multiple readers with different experience knee MRI evaluation, in the assessment of meniscal signal, morphology and position.

## Material and methods

### Patients

MRI scans of 96 patients were retrospectively reviewed between 21 January and 30 March 2020. The main indications for knee MRI examination were persistent or post-traumatic knee pain. Of these patients, only those that met the following criteria were selected: no surgery or arthroscopy prior to the examination, age between 18 and 90 years, no signs of advanced gonarthrosis (exposure of the subchondral bone, osteophytotic deformation of the joint heads).

### MRI technique

All MRI examinations were performed using a 1.5 T unit (Avanto, Siemens) with dedicated knee coil. Each patient was placed in supine position with flexed knee. The flexion angle was approximately 15°–20° in all examinations. Details about the MRI protocol are summarized in Table 1.

**Table 1:** MRI sequences used to evaluate meniscal position, morphology and signal intensity

	T1 TSE sag	Spair sag/cor	T2 TSE axial
TR (time to repeat) (ms)	400	3200	4330
TE (time to echo) (ms)	14	32	81
Matrix	240 × 320	240 × 320	358 × 448
Thickness (mm)	3	3	3
Number of signal averages (NSA)	1	1	2

Before starting the MRI examination, all patients received information and explanation of relative and absolute contraindications to MRI and informed consent was obtained. The MRI images were finally evaluated using a dedicated workstation.

### Image analysis

Three radiologists with 15, 8 and 3 years of experience in musculoskeletal imaging (respectively EAG, LC, MC) reviewed MRI images using a Picture Archiving and Communications System (PACS). Each radiologist was blinded to the original report of knee MRI. The studies were randomly sorted from all sessions and sets of MRI images included in the study.

The parameters assessed in each MRI were the meniscus morphology, its position and the presence of areas of altered signal in the context of the meniscus.

The morphology of the meniscus was evaluated mainly in coronal and sagittal SPAIR sequences, considering changes in dimensions and shape (Fig. 3). The meniscus position was assessed principally in coronal SPAIR sequences, in relation to the margin (inner or outer) of the tibial plateau, considering a possible meniscal extrusion from the joint plane (Fig. 4).

Finally, the altered signal of meniscus was evaluated in sagittal, coronal and axial SPAIR sequences, as signal increasement seen in at least two slices. This was considered pathologic when it involved at least one joint side (type III lesion) (Fig. 2).

Each radiologist filled a standard form and assigned a score of “1” or “0” for each parameter: “1” if the findings were considered pathological, “0” if the study was considered physiological. The score was awarded after fully viewing all the sequences included in each study in the three orthogonal planes.

### Statistical analyses

Cohen's Kappa (k) statistics were performed to evaluate the inter-observer agreement; a κ value less than or equal to 0.20 indicated poor inter-observer agreement, a κ value of 0.21–0.40 indicated fair agreement, a κ value of 0.41–0.60 indicated moderate agreement, a κ value of 0.61–0.80 indicated good agreement, and a κ value of 0.81–1.00 indicated excellent agreement. Statistical significance was set at *p* < 0.05. The data analysis for this paper was generated using the Real Statistics Resource Pack software (Release 7.2). Copyright (2013–2020) Charles Zaiontz. www.real-statistics.com.”

## Results

**Fig. 1:**
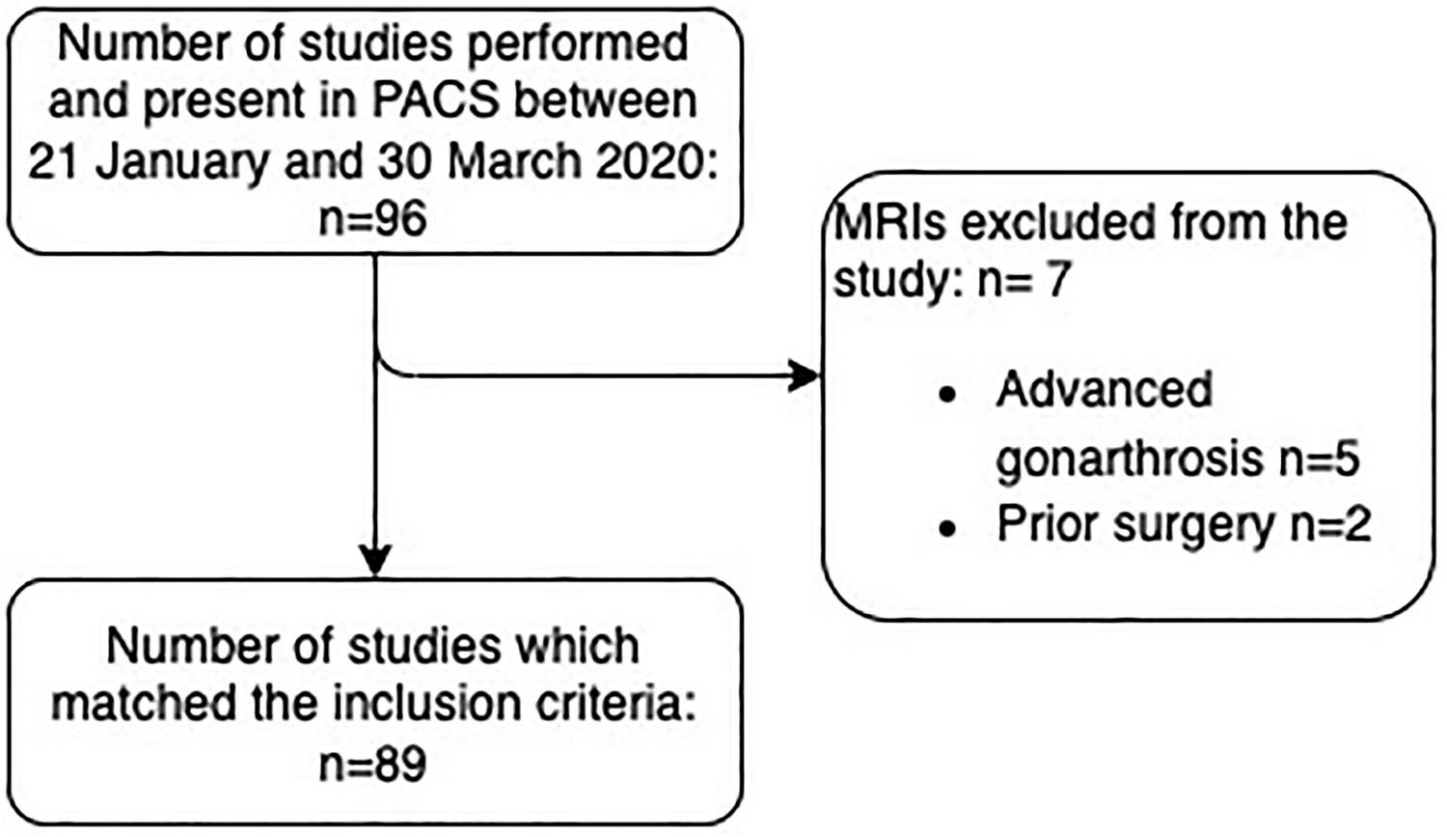
Selection of studies included in the research

**Fig. 2:**
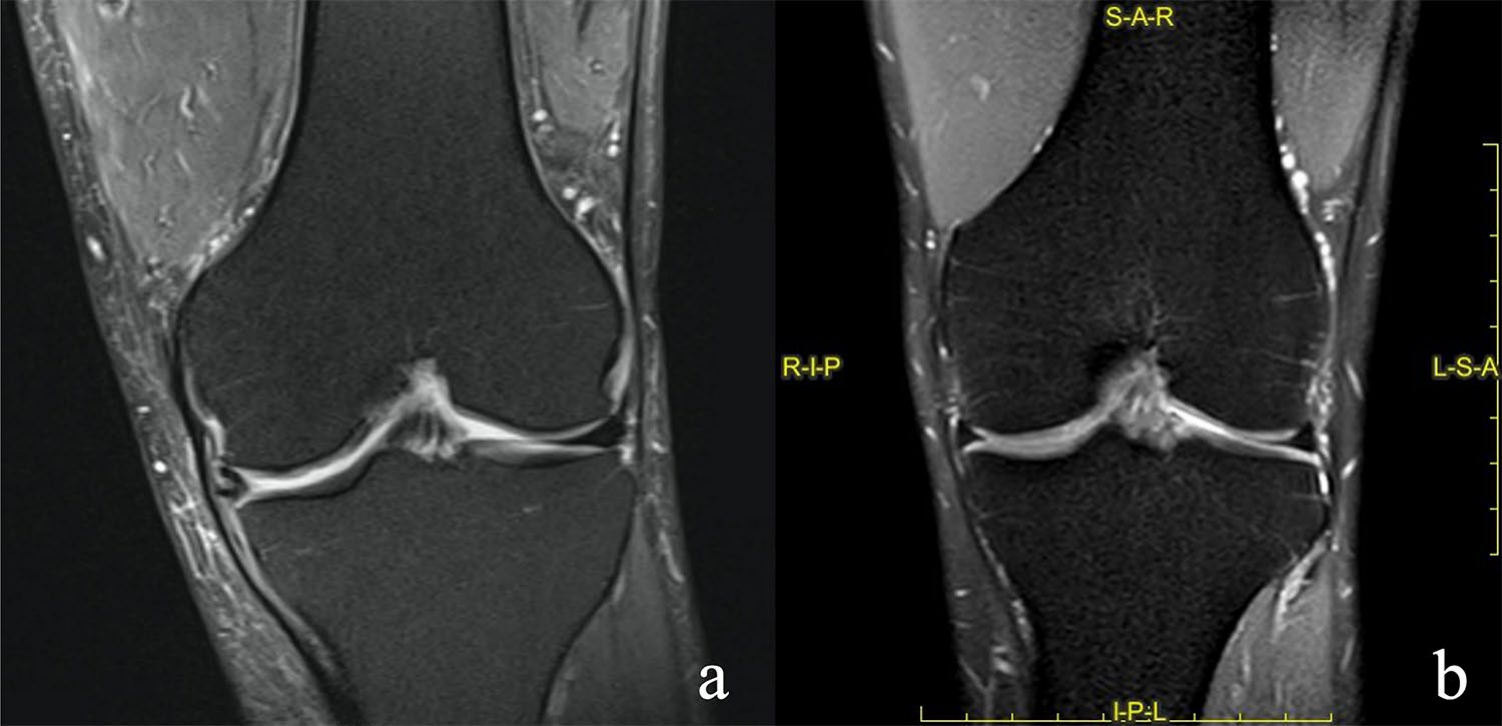
**a, b** Coronal SPAIR sequences (**a**, **b**). **a** Abnormal signal of the external meniscus, in this case the signal was clearly abnormal, and the interpretation achieved a good grade of concordance. **b** an example of a “physiological” examination

Eighty-nine MRI dataset matched the inclusion criteria (Fig. 1). Fifty-three men and thirty-six women were included with an age range of 18–84 years (mean age 59 years) (Fig. 2).

The results are summarized in Tables 2, 3 and 4.

**Table 2:** *k* values relating to the meniscus position assessment

	Position *k* value assessment					
	MM R1	MM R2	MM R3	LM R1	LM R2	LM R3
MM R1		0.603	0.57			
MM R2	0.603		0.871			
MM R3	0.57	0.871				
LM R1					0.715	0.283
LM R2				0.715		0.572
LM R3				0.283	0.572

**Table 3:** *k* values relating to the meniscus signal assessment

	Signal *k* value assessment					
	MM R1	MM R2	MM R3	LM R1	LM R2	LM R3
MM R1		0.876	0.754			
MM R2	0.876		0.877			
MM R3	0.754	0.877				
LM R1					0.867	0.692
LM R2				0.867		0.761
LM R3				0.692	0.761

**Table 4:** *k* values relating to the meniscus morphology assessment

	Morphology *k* value assessment					
	MM R1	MM R2	MM R3	LM R1	LM R2	LM R3
MM R1		0.859	0.504			
MM R2	0.859		0.534			
MM R3	0.504	0.534				
LM R1					0.673	0.323
LM R2				0.673		0.462
LM R3				0.323	0.462	

**Fig. 3:**
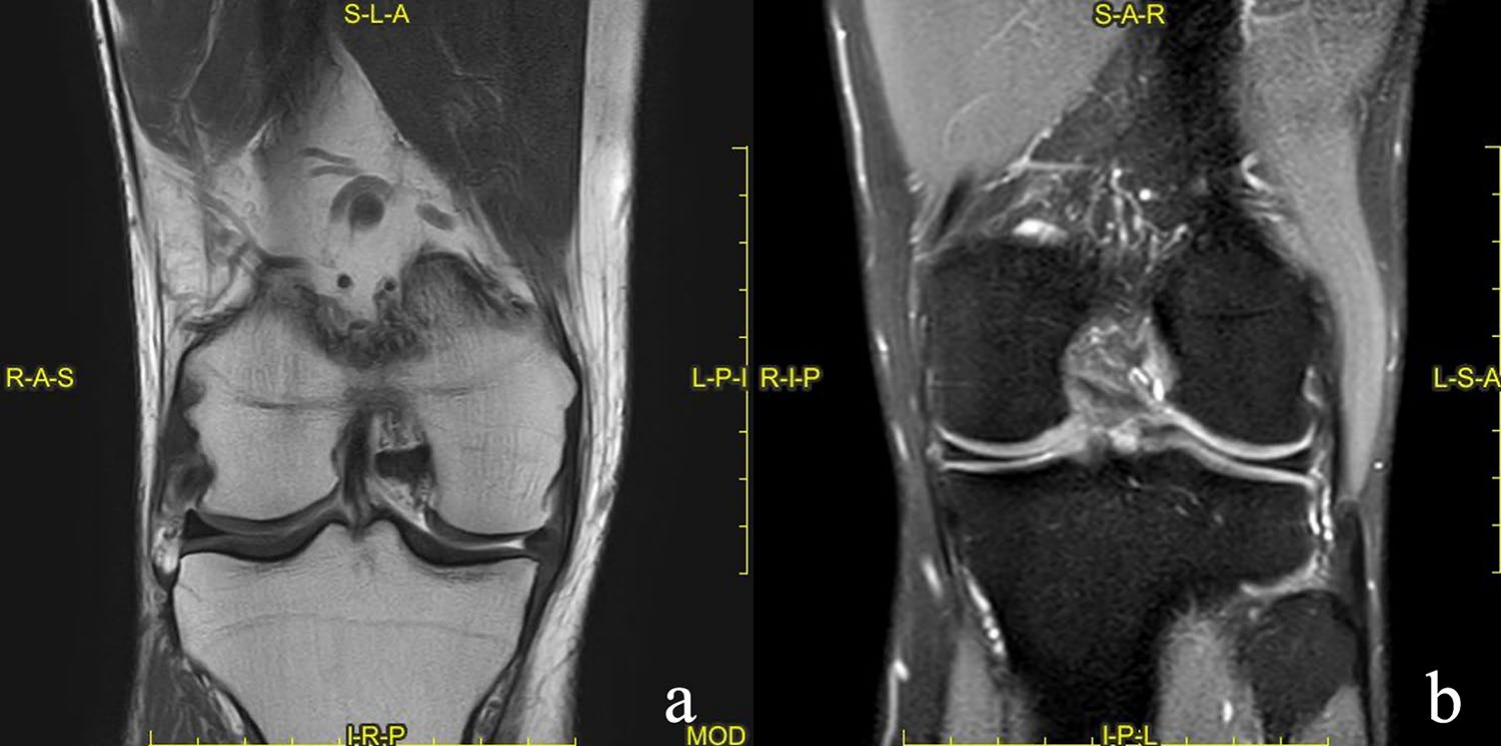
**a, b** Coronal T1 (**a**) and SPAIR sequences **b**. **a** Abnormal morphology of the internal meniscus, in this case the morphology was clearly abnormal, and the interpretation achieved a good grade of concordance. **b** an example of a “physiological” examination

**Fig. 4:**
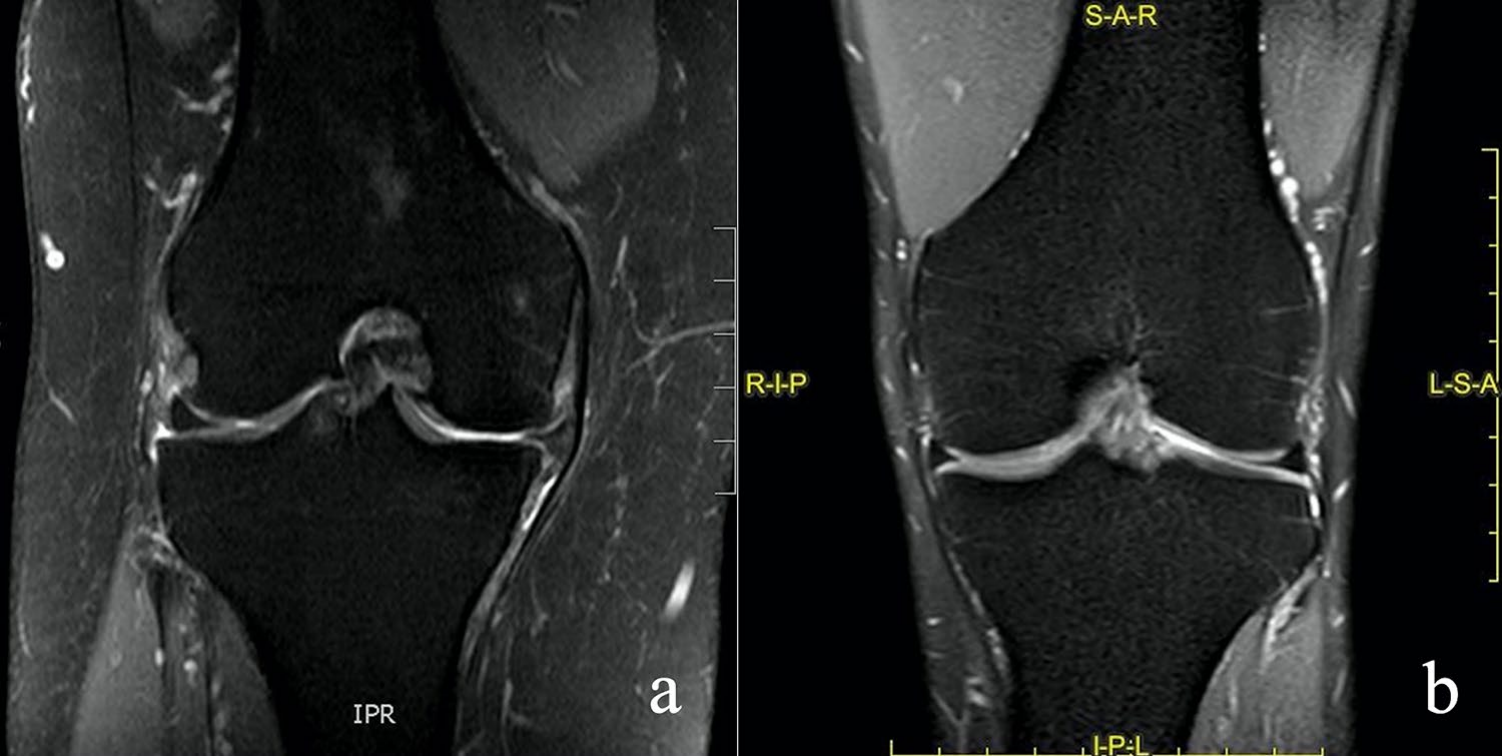
**a, b** Coronal SPAIR sequences. **a** Abnormal location of the internal meniscus that is extruded, the degree of dislocation interpretation in this case was homogeneous achieving a good grade of concordance. **b** an example of a “physiological” examination

The best results of concordance were found in the signal assessment with k values up to 0.877 (Fig. 4), while the morphology and position showed suboptimal results in particular at the lateral meniscus (whereas in the medial meniscus the results are better with a Cohen k range between 0.504 and 0.809) (Figs. 3 and 4).

## Discussion

The study of a pathological condition using a diagnostic test based on subjective findings must be validated based on the reproducibility of results. The readers' agreement level with a certain diagnostic parameter makes possible to ascertain how reliable it is in describing a pathology and how much experience influences image interpretation.

For example, ultrasound (US) is a very useful and often extremely accurate diagnostic tool for some diseases. However, the poor reproducibility due to the strong dependence on operator experience makes it an unsuitable method for describing subjective parameters. In fact, the level of agreement between operators with different experience is often very low [15–17].

MRI is a diagnostic method with a high level of inter-individual concordance, so that the description of some parameters does not appear to be significantly dependent on operator experience. [18, 19].

In our study, we analyzed the concordance level between three operators with various experience in reading knee MRI images describing different parameters of meniscal pathology. A thorough understanding of the imaging protocols, meniscal anatomy, surrounding anatomic structures and anatomic variants and pitfalls is critical to ensure diagnostic accuracy and prevent unnecessary surgery. Awareness of common diagnostic errors can ensure accurate diagnosis of meniscal tears [20, 21].

To the best of our knowledge, inter-individual concordance in the assessment of menisci between radiologists with different levels of experience has never been tested.

The parameter with the worst level of agreement was the position of the lateral meniscus in relation to the tibial plateau (*k* = 0.49).

The reason could be the variability of the anatomical landmark used by each radiologist to describe the degree of extrusion and the variability of meniscal position in relation to different knee flexion angles.

Meniscal extrusion may be underestimated on supine MRI scans, instead, ultrasound is a dynamic study and can detect the knee joint line, the presence of extrusion and is able to a better quantification of the condition compared with MRI [22].

A similarly poor result was obtained by Jones LD et al. [23] where the low reproducibility degree was apparently due to the different landmarks used to keep the knee in the correct position during image acquisition. The same authors have also demonstrated that analyzing meniscal extrusion using only coronal MRI datasets overestimates the true extrusion degree of the medial meniscus [23].

The evaluation of meniscus extrusion, using the medial tibial spine as the only anatomic reference, demonstrates the true meniscus displacement from the tibial rim and may somewhat restrict the variability of interpretations [23]. Also De Smet A et al. suggest assessing the meniscal extrusion degree in coronal MRI slices by measuring the distance between the meniscal body and the apex of the medial tibial spine [20]. Physicians and researchers should consider this result while conducting longitudinal studies evaluating meniscal stability over time or following surgery.

The evaluation of meniscal morphology also achieved suboptimal concordance values (average *k* = 0.56), particularly regarding the morphology of the lateral meniscus (average *k* = 0.49).

The reasons for the low diagnostic accuracy of the lateral meniscus include: the presence of various local anatomical structures in the area of the attachment point of the posterior root, the frequent association with an anterior cruciate ligament injury, the presence of artifact of popliteal pulse and a greater length of the posterior root of the lateral meniscus than the internal one, increasing the angle between long axis and coronal plane [24].

MRI is currently the gold standard method to detect meniscal lesions and to thoroughly describe meniscal morphology [1]. Nevertheless, inter-individual anatomical variability sometimes makes it complex to classify particular variants as a source of pathology when they may be asymptomatic anatomical variants. These considerations could explain the poor reproducibility of this parameter.

The measure that achieved the best inter-individual concordance was the evaluation of signal intensity. The high level of agreement may be due to the presence of an almost universally accepted and applied classification of meniscal injuries [1, 21].

In particular, 94% of the lesions described as type III, where the altered signal intensity reaches the joint plane, were confirmed by arthroscopic evaluation, and the presence of this sign became the MRI standard diagnosis of a meniscal injury [25].

According to our results, we can conclude that signal intensity is an almost universally acceptable parameter, regardless of the reader's experience.

To decrease interpersonal variability in the study of meniscus position and morphology, a step-by-step assessment of the examination should be done. The examination should start from the study of meniscus morphology, remembering all the possible congenital and degenerative alterations. Then it should move on to the evaluation of meniscus position by taking reference points to standardize the degree of meniscal extrusion and finally it should pass to evaluation of meniscus intensity signal, index of meniscal injury.

Furthermore, each of these evaluations should be made on all three planes, coronal, sagittal and axial. However, there were several limitations in this analysis: the MRI technique used to evaluate meniscal position, morphology and signal intensity was always the same with the same sequences and protocol (TR, TE, matrix, thickness), also the presence of patient movement artifacts could be considered a limitation of our knee joint MRI examination. Another one can be found in the different inclusion and exclusion criteria for patients: those with previous surgery or arthroscopy were removed from our study. The degree of extrusion and morphology, on the other hand, are subject to interpretation and depend on the radiologist's level of experience and knowledge of meniscal pathology.
